# Long term conjugated linoleic acid supplementation modestly improved growth performance but induced testicular tissue apoptosis and reduced sperm quality in male rabbit

**DOI:** 10.1371/journal.pone.0226070

**Published:** 2020-01-10

**Authors:** A. M. Abdelatty, O. A. M. Badr, S. A. Mohamed, M. S. Khattab, SH. M. Dessouki, O. A. A. Farid, A. A. Elolimy, O. G. Sakr, M. A. Elhady, G. Mehesen, M. Bionaz

**Affiliations:** 1 Department of Nutrition and Clinical Nutrition, Faculty of Veterinary Medicine, Cairo University, Giza, Egypt; 2 Department of Genetics and Genetic Engineering, Faculty of Agriculture, Benha University, Qalyubia, Egypt; 3 Department of Pathology, Faculty of Veterinary Medicine, Cairo University, Giza, Egypt; 4 Department of Animal Production, Faculty of Agriculture, Cairo University, Giza, Egypt; 5 Department of Physiology, National Organization for Drug Control and Research, Giza, Egypt; 6 Department of Pediatrics, University of Arkansas for Medical Sciences, Little Rock, AR, United States of America; 7 Arkansas Children’s Nutrition Center, Little Rock, AR, United States of America; 8 Department of Animal Production, National Research Centre, Giza, Egypt; 9 Department of Toxicology and Forensic Medicine, Faculty of Veterinary Medicine, Cairo University, Giza, Egypt; 10 Department of Animal and Rangeland Sciences, Oregon State University, Corvallis, OR, United States of America; University of Hyderabad, INDIA

## Abstract

Conjugated linoleic acid (CLA) is known for its multiple benefits including improvement of growth, increasing lean mass, and anti-carcinogenic effects. However, when used in long-term supplementations CLA does not improve semen parameters in boar and bull and reduces fertility in Japanese quails. The content of unsaturated fatty acids in dietary lipids plays a significant role in spermatogenesis owning the high proportion of unsaturated fatty acids in plasma membrane of sperms. Whether CLA plays a role in testicular tissue and epididymal fat is still unknown. Therefore, in this study we hypothesize that long-term supplementation of equal proportion of CLA isomer mix (*c*9,*t*11-CLA and *t*10,*c*12- CLA) in rabbit bucks might alter male reproductive potentials. Twelve V-Line weaned male rabbits were used in 26 weeks trial, rabbits were individually raised and randomly allocated into three dietary groups. Control group (**CON**) received a basal diet, a group received 0.5% CLA **(CLA 0.5%)**, and a group received 1% CLA **(CLA 1%).** Rabbits were euthanized at the end of the trial and several parameters were evaluated related to growth, semen quality, and testicular and epididymal tissue histopathology and transcriptome. The long-term supplementation of CLA increased feed intake by 5% and body weight by 2–3%. CLA 1% decreased sperm progressive motility. In testicular tissue L-carnitine and α-tocopherol were decreased by CLA supplementation. In epididymal fat, CLA tended to decrease concentration of polyunsaturated fatty acids, the expression of *SCD5* gene was upregulated by CLA 1% and *CASP3* gene was upregulated by CLA 0.5%. Transcription of *PPARG* was downregulated by CLA. Feeding 1% CLA also decreased testicular epithelial thickness. Long-term supplementation of CLA modestly enhanced male rabbit growth, but negatively impacted male reproduction, especially at high dose of CLA.

## Introduction

The discovery of anticancer properties made the conjugated linoleic acid (CLA) top-studied fatty acid by the scientific community [1]. Multiple studies were conducted to investigate the beneficial effects of CLA, which include enhancement of immune function and decrease inflammation in several animal models [2,3]. The nutrigenomic effects of CLA have been studied to evidence the promising nutritional properties of CLA in animal diets [4]. Additionally, the positive effect of CLA on weight loss by shifting the energy repartition in the body could explain its wide use as male dietary supplement among young athletes [5].

Despite the above-mentioned beneficial effects of CLA and the wide use of it as a dietary supplement in human and animals, the effects of long-term supplementation of CLA on body weight and male reproduction are very limited. Studies conducted to evaluate the effects of CLA on male reproduction were mostly short-term (i.e., less than 6 month) [6] and conducted after puberty looking at the effect of CLA on semen parameters either through dietary supplementation [7] or added directly to semen to extend cryopreservation [8]. Some studies reported that CLA supplementation increased fertility [9] while other studies have shown no [7,10] or limited effect on fertility based on semen evaluation only [11]. In a study on Japanese quails, CLA reduced fertility and hatchability [12].

Rabbit have high reproductive rate compared to other livestock. According to FAO [13], one rabbit buck could inseminate 15 rabbit does. Therefore, for future sustainability of rabbit production, male reproductive potential of rabbit bucks is of major concern. On the other side, dietary lipid plays a crucial role in sperm plasma membrane formation, particularly polyunsaturated fatty acids (PUFA) [14]; therefore, CLA was added in the rabbit diet to investigate whether it plays a positive or negative role in rabbit fertility. To the authors' knowledge, the effects of long-term CLA supplementation on growth and male reproductive organs in male rabbit have not been investigated. Therefore, this study hypothesized that long-term dietary CLA supplementation enhances growth but has a negative impact on male reproduction in rabbit bucks. For this purpose, semen of rabbits was evaluated and testicular and epididymal tissue samples were subjected to histopathological and transcriptomic assessments.

## Materials and methods

### Ethical approval

The experimental procedures for this study approved by Cairo University Institutional Animal Care and Use Committee (approval # CU/II/F/95/18). Animal number was kept to the minimum and euthanasia was carried out in accordance with AVMA (American Veterinary Medical Association) guidelines. Rabbits were purchased from the rabbit unit of the Faculty of Agriculture, Cairo University, and handled with care. Due to limited number of rabbits used in the study, rabbits were raised individually, and samples were analyzed in technical triplicates for all samples collected after euthanasia.

### Experimental design, animals housing, and diet

Experimental diets were formulated to meet or exceed the NRC (National Research Council) requirements for growing rabbit [15]; 2500 Kcal digestible energy/ Kg diet, 16% crude protein, fat 2%, and crude fiber 10–12% (Table 1). Twelve V-line strain weaned male rabbits (35 d ± 5) were used in a 26 weeks experiment. Rabbits were blocked for body weight 605 g (±33.5; *P* = 0.99) and randomly allocated into three iso-nitrogenous-iso-caloric dietary treatments (*n* = 4/group) as follows: 1) **CON** group fed basal diet supplemented with 1% oleic acid (Techno Pharmachem, India), 2) **CLA 0.5%** group was fed on diet supplemented with 0.5% CLA (Lutrell Pure; BASF, Ludwigshafen, Germany; certified to contain equal proportion of *c*9,*t*11- and *t*10,*c*12-CLA; EFSA, 2016) plus 0.5% oleic acid, and 3) **CLA 1%** group was fed a diet supplemented with 1% CLA. Pure oleic acid was used to balance the diet for energy. Linoleic acid was not selected to balance the diet as linoleic acid was found to increase tissue accumulation of CLA contents as noticed by Garcia et al. [16].

**Table 1:** Diet ingredients^1^.

Ingredient	(g/kg)
Barley	230
Soybean meal (44%)	160
Clover hay	300
Wheat Bran	263
Molasses	30
Limestone	10
Sodium Chloride	4
Premix^2^	3
Oleic acid^3^ CLA^4^	Variable Variable

^1^diet was formulated to provide 2500 Kcal digestible energy/kg diet according to rabbit NRC, 1977.

^2^The premix provides the following (per kg diet): 15,000 IU of Vit. A; 100 mg Vit. E; 21 mg Vit. K3; 10 mg Vit. B_1_; 40 mg Vit. B_2_; 15 mg Vit. B_6_; 0.1 mg Vit. B_12_; 200 mg Niacin; 100 mg Pantothenic acid; 0.5 mg Biotin; 10 mg Folic acid; 500 mg Choline Chloride; 450 mg Zn; 600 mg Mn; 0.3 mg Fe; 50 mg Cu; 250 mg I.

^3^Oleic acid was added at a rate of 1% on the control group diet (CON), and 0.5% in the CLA 0.5% group diet.

^4^Dietary CLA (Conjugated linoleic acid) was added at a dose of 0.5% in the CLA 0.5% diet and 1% in the CLA 1% diet.

Feed samples were collected from each dietary treatment and analyzed (**Table 2**) for chemical composition according to AOAC [17]. Rabbits were individually raised in semi-closed system and had free access to water *via* nipple system. Individual body weight and feed intake were recorded weekly.

**Table 2:** Chemical composition of the experimental diets (g/kg DM).

Item (%)	CON^1^	CLA 0.5%^2^	CLA 1% ^3^
Dry matter	911.70	915.00	915.90
Crude protein	186.20	183.80	183.30
Crude fat	43.40	41.30	41.00
Ash	135.2	130.00	129.40
Crude fiber	154.40	147.50	145.50

^1^Control diet supplemented with 1% oleic acid.

^2^Diet supplemented with 0.5% CLA+0.5% oleic acid.

^3^Diet supplemented with 1% CLA.

### Semen collection and sperm kinetics

Due to the large variability on the moment of puberty in rabbits (19 to 24 weeks) [18,19] semen was collected twice a week for 7 successive weeks starting from week 20 of the experiment. Semen was collected from the bucks by using an artificial vagina according to Viudes-de-Castro et al., [20]. The ejaculates were transferred immediately to the laboratory and maintained in a water bath (38.5 ºC) until evaluation. An aliquot from each sample (10 μL) was diluted 1:20 in Tris–citrate–glucose extender (250 mM tris-hydroxy methyl amino methane, 83 mM citric acid, 50 mM glucose, pH 6.8 to 7.0) and subjected to motion characteristics evaluation using a computer-assisted sperm analysis system (CASA; instrument SpermVision^™^ software minitube Hauptstraße 41. 84184 Tiefenbach, Germany). Ten microscopic fields were captured for each sample and the average of motility parameters were recorded including DAP (Distance Average Path, μm), DCL (Distance Curved Line, μm), DSL (Distance Straight Line, μm), VAP (Velocity Average Path, μm/s), VCL (Velocity Curved Line, μm/s), VSL (Velocity Straight Line, μm/s), STR (Straightness Track, VSL/VAP,%), LIN (Linearity, VSL/VCL,%), WOB (Wobble VAP/VCL,%), ALH (Amplitude of Lateral Head Displacement, μm), and BCF (Beat Cross Frequency, Hz).

### Sperm concentration and morphology

Sperm concentration and morphology were evaluated for all samples according to Perumal et al., [21]. All chemicals used for semen processing were purchased from Sigma-Aldrich (Cairo, Egypt) unless otherwise stated.

Sperm concentration was determined using a hemocytometer and expressed as 10^6^ sperm/ml. The proportion of live to dead spermatozoa was estimated using Eosin-Nigrosin stain. Sperm suspension smears were prepared by mixing 10 μL of semen sample with 20 μL of stain on a warm slide then allowed to dry at room temperature. Viability was assessed by phase-contrast microscope at a magnification of 1000× using immersion oil. Only sperm showing strict exclusion of stain were counted as viable, while sperm displaying partial or complete purple staining were considered non-viable. In the same fields of eosin-nigrosin stained slides, the number of spermatozoa with abnormal head morphology, cytoplasmic droplets and abnormal tails were also counted (200 sperm per slide) to determine the percentage of morphological abnormality.

For assessment of acrosomal integrity, a drop of semen was smeared on a pre-warmed slide. The slide with the drop was allowed to dry in a flow of air followed by immersion in buffered formal saline for 15 min, washed for 15–20 min, dried and immersed in the buffered Giemsa solution for 90 min then rinsed in distilled water, and allowed to dry.

The functional integrity of the sperm plasma membrane was evaluated by hypo-osmotic swelling test (HOST). The HOST assay was performed by mixing 20 μL of semen with 200 μL of a hypo-osmotic solution. The mixture was incubated for 60 min at 37° C and then a drop was smeared on a slide. A minimum of 200 sperm was observed under phase contrast microscope at 400× magnification and the percentages of sperm with swollen and curled tails were recorded for each sample.

### Euthanasia and blood sampling

Rabbits were euthanized using Ketamine and Xylazine protocol as described in Simón et al., [22] and in accordance with the AVMA guidelines. Blood was collected after euthanasia for serum separation. Blood samples were allowed to clot for 20 minutes. The clot was removed by centrifugation at 2,000 ×g for 10 minutes. The serum was separated and stored at -20 ºC till analysis [23].

### Serum testosterone and testicular metabolites

A testosterone ELISA kit (Cat No. 582701, Cayman Chemicals, USA) was used for serum testosterone determination according to the manufacturer instructions. The level of testosterone in serum was presented as ng/ml.

Testicular oxidative stress metabolites malondialdehyde (MDA), 8-hydroxy-2-deoxyguanosine (8-OHDG), L-carnitine and α-tocopherol were assessed with HPLC (Agilent HP 1200 series; USA). The analytical column was Supelcosil C18 (5 μm particle and 80 Ao pore size) (250 × 4.6 ID). Testicular MDA level (nmol/g tissue) and the 8-OHDG expressed as pg/g tissue were determined as described by Ahmed-Farid et al., [24]. L-carnitine concentration was detected as described by Alcorn et al., [25] and vitamin E (α-tocopherol) was determined by the method of McMurray et al., [26].

### Epididymal fatty acid profile

Epididymal fatty acids were extracted according to Abdelatty et al., [27], briefly, lipids were extracted with chloroform: methanol mixture (2:1), a standard fatty acid mixture was purchased from Sigma‐Aldrich (Sigma, St. Louis, MO). Fatty acids were determined according to O'fallon et al., [28]. Gas chromatography (GC) was done with an Agilent 7890A. Hydrogen was the carrier gas with a column head pressure of 10 psi. Total flow rate at the split vent was 50 ml/min, the flow rate through the column was 2.5 ml/min and the septum purge was 2.5 ml/min. One μl was injected with the injector set at 220°C using the split-less injection mode. The temperature gradient started with a 70°C initial temperature, a linear increase to 170°C at a rate of 20°C/min, a slower linear increase to 170°C at a rate of 0.8°C/min to separate closely eluting fatty acids, followed by an increase to reach 220°C at 20°C/min, and a final 2.5 min hold. The total run time was 20.1 minutes. Fatty acid desaturation indices were calculated according to Sirriet al., [29].

### Histomorphometry

Tissue samples from testis and epididymis were collected immediately after euthanasia and fixed in 10% neutral formalin buffer. Tissue samples were then processed using the paraffin embedding technique and sectioned using a microtome (Leica 2135, Germany) into 3–5 micron sections. Tissue sections were then stained with routine hematoxylin and eosin stain and Masson’s trichrome stain. Fixed epididymal tissue samples were also sectioned using cryostat in order to stain fat using Sudan Black stain. Stained tissue sections were examined using a light microscope and photographed (Olympus XC30 camera, Japan). The thickness of the epithelium lining seminepherous tubules was measured from the basement membrane to the lumen in 10 tubules/testis at angle of 90 degrees using TS view software in order to calculate the mean of epithelial thickness.

Histopathological changes were evaluated using Johnsen's scoring system in 10 seminepherous tubules in each testicular tissue. Score 1 indicated no seminiferous epithelium; Score 2: presence of Sertoli cells only and no germinal cells; Score 3: presence of spermatogonia only; Score 4: few spermatocytes with no spermatozoa or spermatids; Score 5: many spermatocytes with no spermatozoa or spermatids; Score 6: few early spermatids with no spermatozoa and no late spermatids; Score 7: many early spermatids with no spermatozoa and no late spermatids; Score 8: few late spermatids and less than five spermatozoa per tubule; Score 9: many late spermatids, disorganized epithelium indicating slightly impaired spermatogenesis; Score 10: full spermatogenesis and perfect tubules [30].

### Immunohistochemistry

Paraffin-embedded tissue sections of testis and epididymis were immunohistochemically stained using caspase-3 primary antibody (Novus Biologicals, USA) as a hallmark of apoptosis. Level of caspase-3 was quantified using avidin-biotin-peroxidase (Novus Biologicals, USA). All procedures were carried out according to the manufacturer protocol. For color development 3,3′-diaminobenzidine stain was used and hematoxylin was used as counterstain. The percent area of positively stained testicular tissue was measured by using Image J software in three fields/testis.

### Transcriptomic analysis of testicular and epididymal fat

#### Extraction of RNA

Samples of right testis and epididymal fat were collected, snap frozen in liquid nitrogen, and stored at -80 ºC till analysis. Samples were then homogenized in 1ml Qiazol (Qiagen, Valencia, CA) by using TissueLyser (TissueLyser LT; QIAGEN; Cat No./ID: 85600). Homogenization was achieved by 5-mm steel bead shaking at 50 Hz for 5 min according to Sun et al., [31]. The homogenate was used for RNA extraction using Qiagen RNeasy lipid tissue mini-kits according to the manufacturer’s instructions (Qiagen, Valencia, CA). Contaminant genomic DNA was removed with a DNase enzyme (RNase-free DNase set, Qiagen, Valencia, CA). RNA was then collected in 30 μL RNase free water and stored at -80 ºC till cDNA synthesis.

The RNA concentration and purity were measured by Nano-Drop 2000C spectrophotometer (Thermo Scientific, USA). The RNA purity (absorbance ratio, A260/A280) for all samples was ≥ 1.9. The integrity of RNA was verified on 2% agarose gel using gel electrophoresis image (Gel Doc. BioRad) according to Norollahi et al., [32].

#### Synthesis of cDNA template

For the cDNA synthesis 300 ng of RNA per 20 μl reaction were used. cDNA was synthesized using SensiFast cDNA synthesis kits (Bioline, UK) according to the manufacturer’s instructions using a thermal cycler (SensoQuest, GmbH, D-37085).

#### Primer designing and quantitative PCR

Primer pairs for selected target and reference genes are summarized in Table 3. Primer pairs not previously published were designed using Primer Express 3, as previously described by Bionaz and Loor [33]. Primer pairs were purchased from Genwez (New Jersey, USA). Desalted lyophilized primers were reconstituted using RNase free water (Invitrogen, USA).

**Table 3:** Gene names, primer sequences, accession#, and product size of the used genes.

Gene^1^	Accession #	Primers sequences (5’→3’)^2^	bp^3^	Reference
*UXT*	XM_008272555	F: GCGGGACTTGCGAAAGGT	100	Designed
		R: AGCTTCCTGGAGTCGTTCAATG		
*RPS15A*	XM_008257787	F: AGCATGGTTACATTGGCGAGTT	100	Designed
		R:CTGATCACTCCGCACTTGTTTAA		
*HPRT1A*	XM_008267147	F: GCAGACCTTGCTTTCCTTGGT	100	[32]
		R: GCAGGCTTGCGACCTTGAC		
*SCD5*	XM_008267676.2	F:CAAGCATCCAGATGTCATCGA R:CGCTGATCTTATAGTACTTTCTCTGGAA	101	Designed
*PPARG*	NM_001082148.1	F:GAGCCTTCCAACTCCCTCATG R:GAAACCCTTGCATCCTTCACA	102	Designed
*FABP4*	XM_002710655.3	F:AGTCAAGAGCATCATAACCCTAGATG	100	Designed
		R:TTTATCGCCCTCCCGTTTTC		
*LPL*	NM_001177330.1	F:CAGGAGAGACTCAGAAAAAGGTGAT	100	Designed
		R:TTGTCGTGGCATTTCACAAAC		
*BAX*	XM_008252361.2	F:CCCGCGAGGTCTTTTTCC	113	Designed
		R:CAGGGCCTTGAGTACCAGCTT		
*BCL2*	XM_008261439.2	F:GGCTGGGATGCCTTCGT	186	Designed
		R:TTTCGTGAACTGTTTGCATATCTG		
*CASP3*	NM_001082117.1	F:GACAGTGGCATCGAGACAGACA R:GAATAGTAACCAGGTGCTGTGGAA	110	Designed
*GPX1*	NM_001085444.1	F:CAGTTTGGGCATCAGGAGAAC R:GCATGAAGTTGGGCTCGAA	94	Designed

^1^UXT, ubiquitously-expressed, prefoldin-like chaperone; RPS15A, Ribosomal Protein S15a; HPRT1A, Hypoxanthine phosphoribosyl transferase 1; SCD5, Stearoyl-CoA desaturase 5;PPARG, Peroxisome Proliferator Activated Receptor gamma; FABP4, Fatty Acid Binding Protein 4; LPL, lipoprotein lipase; BAX, BCL2 Associated X; BCL2, B-cell lymphoma 2; CASP3, Caspase 3; GPX1, Glutathione Peroxidase 1.

^2^Underline denotes exon-exon junction.

^3^Product size; base pair.

The cDNA was diluted 1:20 using molecular grade RNase free water (Invitrogen, USA). Quantitative PCR reaction was performed using Maxima SYBR Green/ROX qPCR master mix (2x) (Thermo Scientific, USA). The reaction was performed in 0.1 ml qPCR strip tubes with optical caps (Gunster Biotech Co., Taiwan). The reaction consisted of 12.5 μL of SYBR green Master Mix, 11 μL of diluted cDNA, and 0.75 μL of each forward and reverse primer pair to reach final volume of 25 μL. Each sample was performed in triplicate. A non-template control (NTC) was run to check for primer dimer and RNA contamination. The reaction was performed in AriaMx Real Time PCR (Agilent Technologies, USA) using a two steps protocol: initial denaturation at 95 ºC for 10 min, then 40 cycles of denaturation at 95 ºC for 15 s followed by annealing/extension at 60 ºC for 60 s. A melting curve protocol was run at the end of the PCR by heating at 95 ºC for 30 s followed by a 65 ºC for 30 s and 95 ºC for 30 s.

Fluorescent amplification curves data was downloaded from the AriaMx PCR System and analyzed using LinReg PCR software [34]. Three reference genes were tested including ubiquitously expressed transcript (*UXT*), small ribosomal protein subunit 15 (*RPS15A*), and hypoxanthine phosphoribosyl transferase 1 *(HPRT1A)*. The reliability of reference genes was determined using NormFinder [35]. The stability value of the combination of the two best reference genes (*UXT* and *RPS15A*) was 0.19. The normalization factor was calculated as the geometrical mean of the two best reference genes and final data of target genes were obtained by dividing the RTqPCR values obtained by LinRegPCR to the normalization factor.

### Statistical analysis

The MIXED procedure of SAS 9.4 (SAS Institute Inc., Cary, NC, USA) was used for repeated measures analysis of feed intake, body weight, and sperm kinetics, viability, and concentration. Both treatment and time and their interaction were considered as fixed factors in the model and rabbit was considered as random effect.

Shapiro-Wilks test was used to evaluate normality for all parameters before performing the statistical analysis to make sure that *P*-value for Shapiro-Wilks test is > 0.05, meaning that the data were normally distributed for the 12 rabbits.

The UNIVARIATE procedure of SAS 9.4 was used for testing the difference between groups of serum testosterone, testicular metabolites, epididymal fatty acids, gene expression, IHC area % testicular lesion scoring and testicular epithelial thickness. Significance was determined at *P* ≤ 0.05 whereas tendencies were determined at *P* ≤ 0.10.

## Results

### Growth performance

**Fig 1:**
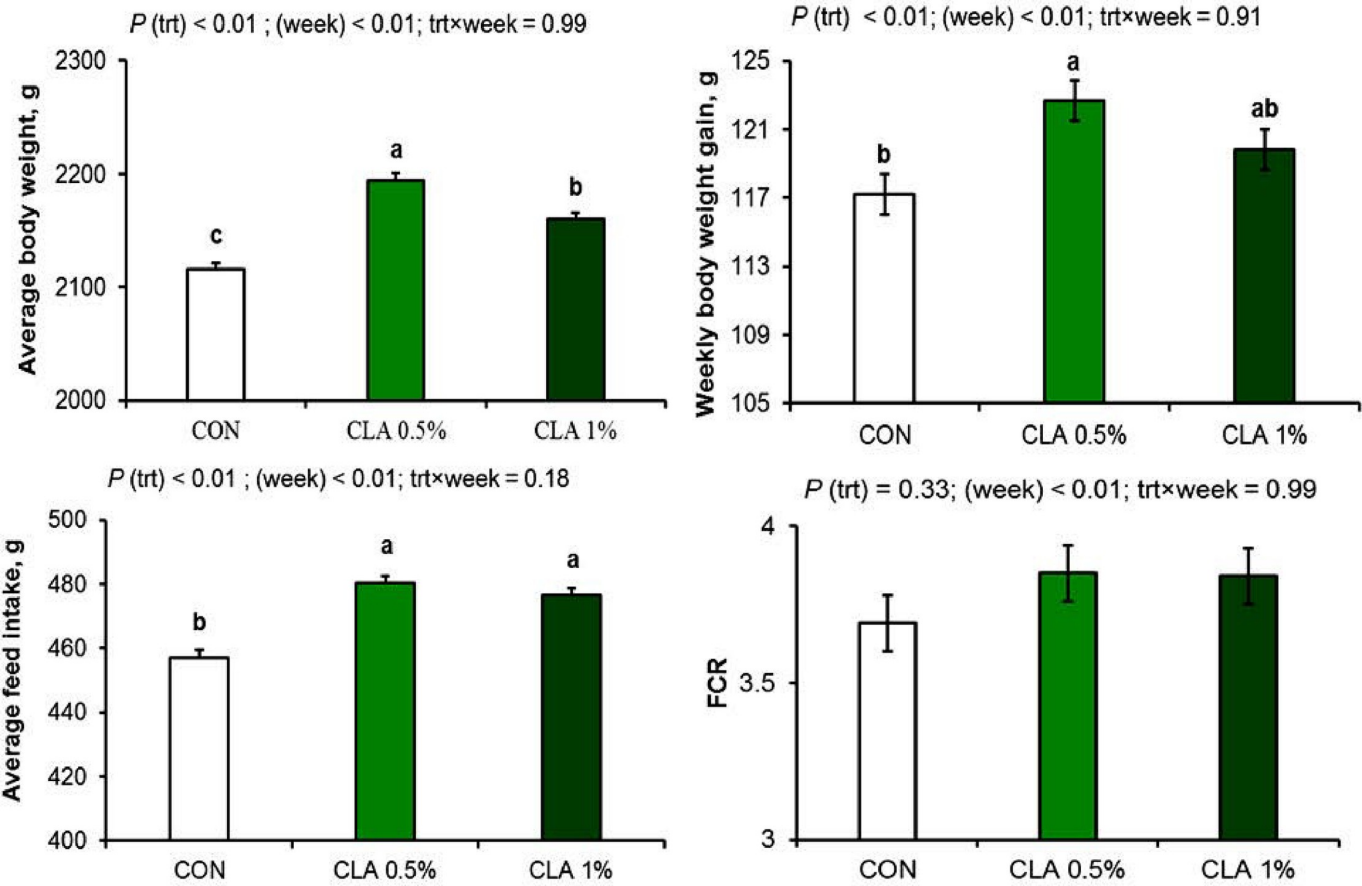
Growth performance of male rabbits with long-term supplementation of CLA. Dietary supplementation of CLA for 26 weeks increased feed intake, body weight, and body weight gain but not the FCR.

The effect of long-term dietary CLA supplementation on growth performance indices is shown in **Fig 1**. Despite the significant increase in feed intake of CLA fed rabbits (*P* < 0.01), the effect of long-term dietary supplementation of CLA on feed intake was minimal, where feed intake increased only by 5% in both CLA fed groups than the control group. Additionally, body weight was significantly affected by CLA supplementation (*P* < 0.01); however, the increase was 2.1% and 3.7% in CLA 1% and CLA 0.5% groups, respectively in comparison to the control group. Additionally, body weight gain was increased only in CLA 0.5% group by 4.6%.

### Sperm Kinetics

Results of sperm kinetics are summarized in **Table 4.** The effect of CLA on sperm kinetics was mostly associated with the dose of 1%, where progressive motility, DAP, and BCF were decreased (*P* ≤ 0.05) by 8.2%, 6.8%, and 6.2%, respectively. The total motile sperm tended to increase by 5.5% in the CLA 0.5% group but not the CLA 1%, compared to the CON group (*P* = 0.07).

**Table 4:** Effect of long term dietary CLA supplementation on sperm kinetics^1^.

Item	CON^2^	CLA 0.5%^3^	CLA 1%^4^	SEM	*P*-value
Motility, %	79.10	84.65	79.57	1.62	0.07
Progressive motility, %	60.52^a^	65.82^a^	52.30^b^	1.78	<0.01
DAP, μm^5^	28.81^a^	29.70^a^	26.86^b^	0.63	0.04
DCL, μm^6^	58.93	57.77	55.35	2.04	0.39
DSL, μm^7^	22.27^ab^	23.30^a^	21.05^b^	0.52	0.05
VAP, μm/s^8^	65.21	67.58	62.74	2.02	0.25
VCL, μm/s^9^	132.51	130.83	125.14	4.73	0.45
VSL, μm/s^10^	50.55	53.16	48.73	1.44	0.13
STR,%^11^	0.77	0.77	0.77	0.01	0.97
LIN,%^12^	0.38	0.40	0.40	0.01	0.54
WOB,%^13^	0.49	0.51	0.51	0.01	0.29
ALH, μm^14^	4.78	4.75	4.77	0.17	0.99
BCF, Hz^15^	31.14^a^	31.05^a^	29.20^b^	0.52	0.05

^1^Values are least square mean, *n* = 4 samples/group, semen was collected twice/week for 7 successive weeks

^**2**^Control group receiving basal diet supplemented with 1% oleic acid

^**3**^Group receiving diet supplemented with 0.5% CLA and 0.5% Oleic acid

^**4**^Group receiving diet supplemented with 1% CLA

^**5**^ Distance average path

^**6**^distance curved line

^**7**^ distance straight line

^**8**^ velocity average path

^**9**^velocity curved line

^**10**^ velocity straight line

^**11**^ straightness track

^**12**^ linearity = VSL/VCL

^**13**^ wobble = VAP/VCL

^**14**^amplitude of lateral head displacement

^**15**^ beat cross frequency.

Different superscript letters denote *P* ≤ 0.05 between treatments.

### Sperm concentration and morphology

**Fig 2:**
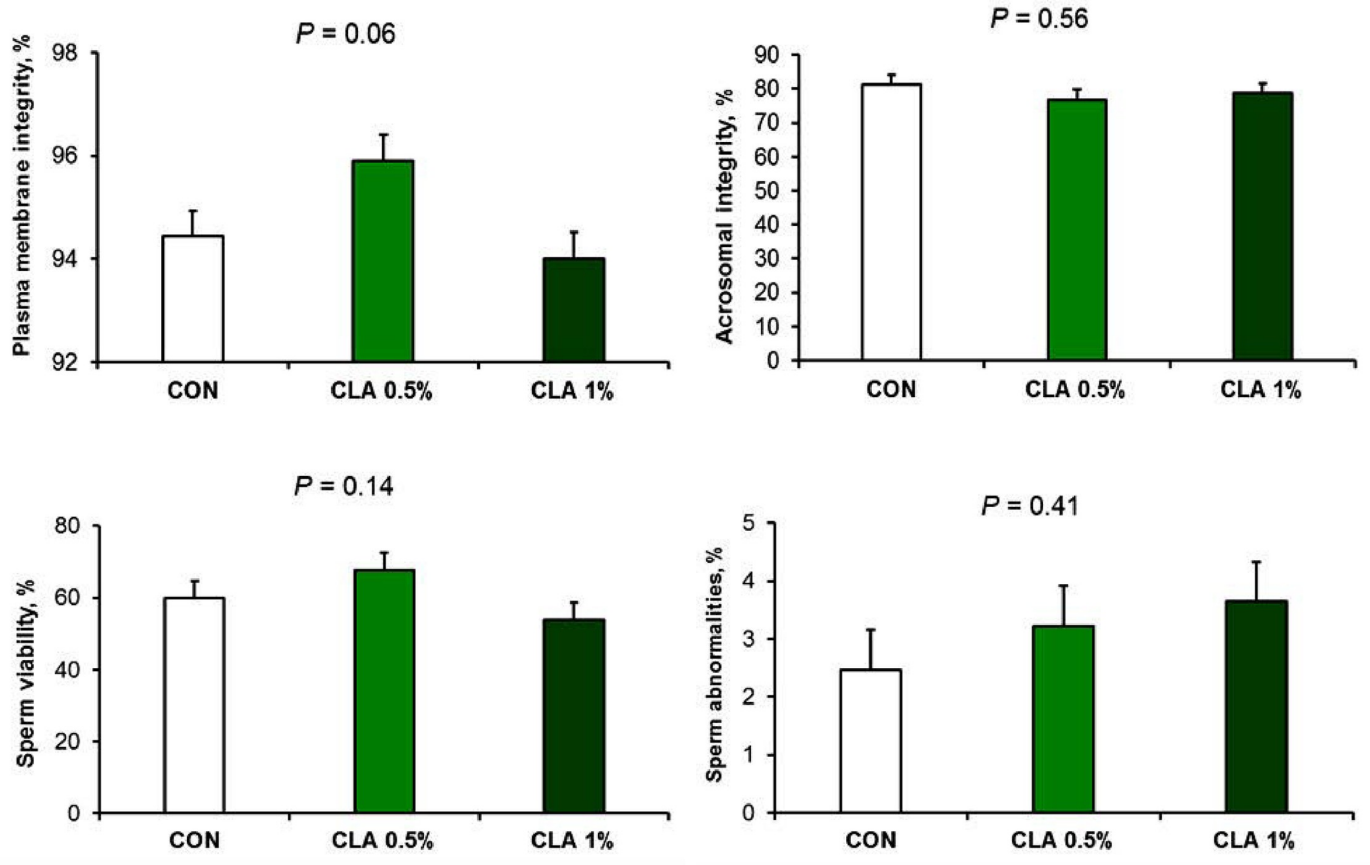
Sperm viability of male rabbits with long-term supplementation of CLA. Long term of dietary CLA supplementation did not improve sperm viability, abnormalities, or acrosomal integrity, however, plasma membrane integrity tended to increase in CLA 0.5% fed rabbits.

**Fig 3:**
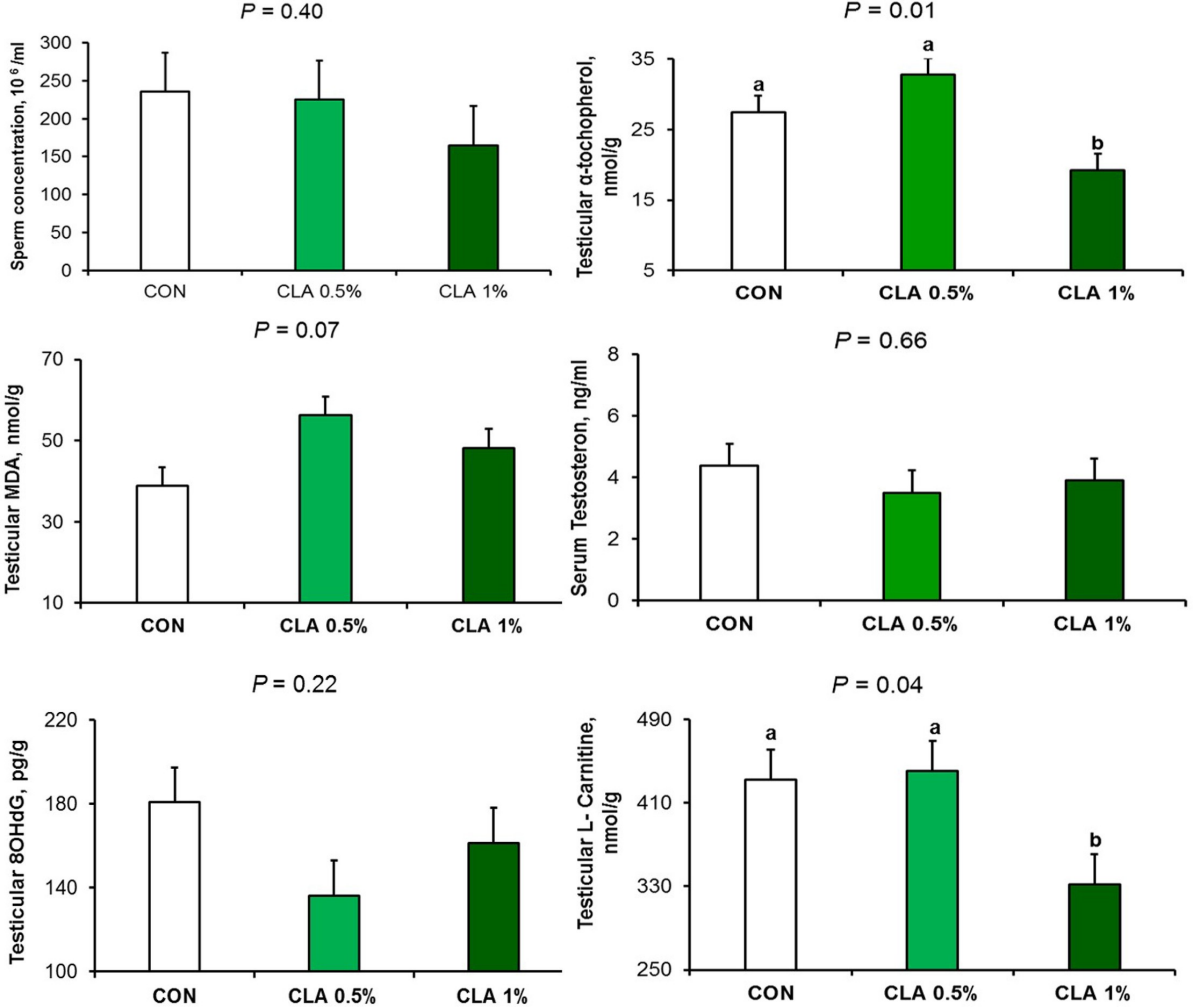
Sperm concentration, serum testosterone, and some testicular metabolites of CLA fed male rabbits. Long term of dietary CLA supplementation did not affect serum testosterone level, or sperm concentration. The level of MDA tended to increase in CLA fed male rabbits. Testicular Tocopherol (Vitamin E) and L-Carnitine were decreased in CLA 1% group only.

Sperm morphology (**Fig 2)** and concentration (**Fig 3**) were not affected by treatments, except for a tendency (*P* = 0.06) for an effect on the plasma membrane integrity due to a numerical higher value in CLA 0.5%.

### Serum testosterone and testicular metabolites

The responses of measured testicular metabolites and serum testosterone level are presented in **Fig 3.** The long-term supplementation of CLA 1% decreased vitamin E (*P* = 0.04) and L-carnitine (*P* = 0.01) compared to CLA 0.5% and CON. Testicular MDA concentration tended to increase (*P* = 0.07) in CLA groups compared to CON.

### Epididymal fatty acid profile

Fatty acid profile of epididymal fat for each group is presented in **Table 5.** Medium-chain fatty acids from C10 to C14 were affected by CLA treatment, with higher values in CLA 0.5% and lower in CLA 1%. Additionally, eicosanoic acid *cis* 11 (C20:1) was decreased by 17.4% by CLA 1% vs. CON. Linolenic acid (C18:3), arachidonic acid (C20:4), and sum of the PUFA tended to decrease in CLA 1% vs. CON. CLA supplementation decreased epididymis total fatty acids in a dose dependent manner (*P* < 0.01).

**Table 5:** Effect of long term dietary CLA supplementation on fatty acid profile and desaturation indices of epididymal fat^1^.

Fatty acid, (mg/g tissue)	CON^2^	CLA 0.5%^3^	CLA 1%^4^	SEM	*P*-value
C10:0	0.60^ab^	0.64^a^	0.53^b^	0.03	0.03
C12:0	0.22^b^	0.27^a^	0.22^b^	0.01	0.02
C14:0	33.09^b^	36.27^a^	30.07^c^	0.75	<0.01
C16:0	279.95	272.46	257.90	16.48	0.64
C16:1	36.35	34.87	32.37	1.75	0.31
C18:0	40.71	39.83	33.68	2.50	0.15
C18:1	67.70	65.20	61.68	3.72	0.54
C18:3	242.11	242.71	196.22	15.22	0.10
C20:0	1.22	1.17	1.07	0.07	0.31
C20:1	3.16^a^	3.01^a^	2.61^b^	0.13	0.04
C20:2	2.47	2.29	2.24	0.13	0.45
C20:4	2.14	2.15	1.78	0.12	0.10
Σ SFA^5^	355.80	350.64	323.46	15.87	0.35
Σ MUFA^6^	107.21	103.08	96.66	4.00	0.23
Σ PUFA^7^	246.72	247.14	200.23	15.12	0.09
Σ Fatty acids	709.73^a^	670.79^b^	620.36^c^	8.54	<0.01
Elongase^8^	0.15	0.15	0.13	0.01	0.62
Δ 9 desaturase (16)^9^	10.66	11.40	11.14	0.50	0.55
Δ 9 desaturase (18)^10^	62.44	61.93	64.76	2.10	0.61
Δ 9desaturase (c16+18) ^11^	22.90	24.41	24.37	1.08	0.53

^1^Values are least square mean, *n* = 4 samples/group.

^2^Control group receiving basal diet supplemented with 1% oleic acid to balance for energy.

^3^Group receiving diet supplemented with 0.5% CLA and 0.5% Oleic acid.

^4^Group receiving diet supplemented with 1% CLA.

^5^ Saturated fatty acids.

^6^ Monounsaturated fatty acids.

^7^ Poly unsaturated fatty acids.

^8^ Elongase = C18:0/C16:0.

^9^ Δ9 desaturase (16) = [C16:1/(C16:1 + C16:0)]*100.

^10^ Δ9 desaturase (18) = [C18:1/(C18:1 + C18:0)]*100.

^11^ Δ9 desaturase (16 18) = [(C16:1 + C18:1)/(C16:1 + C16:0 + C18:1 + C18:0)]*100.

Different superscript letters denote *P* ≤ 0.05 between treatments.

### Histomorphometry findings

**Fig 4:**
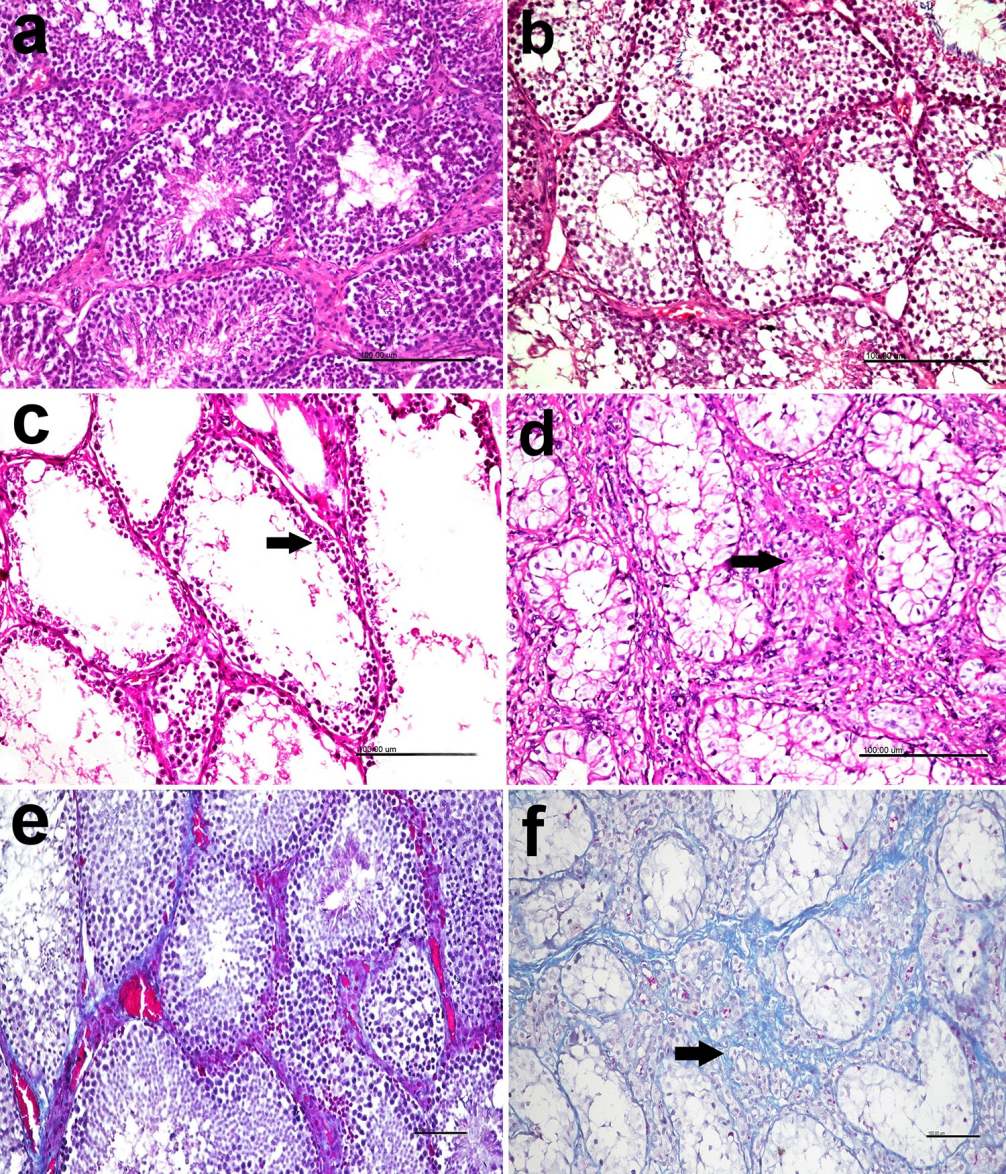
Effect of long term CLA supplementation on testicular tissue of rabbit. Normal histological structure in CON group (4a) Vacuolation of Sertoli cells and decreased spermatogenesis in CLA 0.5% group (4b). Absence of spermatogenesis with few spermatogonial cells lining seminiferous tubules (4c). Testicular degeneration and thickening of interstitial tissue (4d) in CLA 1% group. Hematoxylin and eosin stain X 200. Thickening of interstitial tissue due to Leydig cell hyperplasia in the group treated with 1% CLA (4e) and (4f). Masson’s Trichrome X 200.

The testis of the CON group had multiple layers of spermatogonia lining the seminiferous tubules **(Fig 4A)**. However, in CLA 0.5% group, there was testicular degeneration, Sertoli cell vacuolations, fewer spermatogonial (germ) cells in seminiferous tubules, and few sperms in the lumen (**Fig 4B**). The same findings were in the CLA 1% group with increase severity of the lesions with areas of seminiferous tubules that lost germ cells (**Fig 4C**). In two rabbits of the CLA 1% group, there was a noticeable increase in the interstitial tissue and interstitial (Leydig) cells hyperplasia (**Fig 4D**) which was confirmed by Masson’s trichrome stain (**Fig 4E and 4F**).

**Fig 5:**
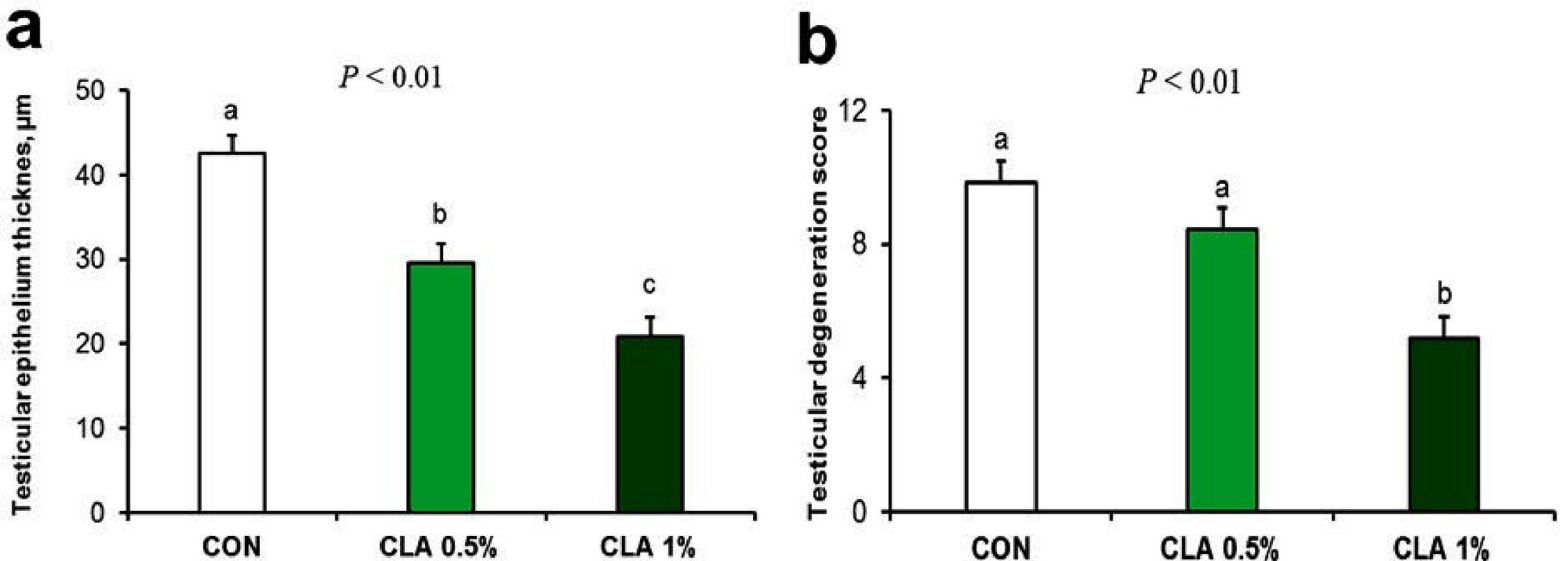
Effect of long-term CLA supplementation on testicular epithelium thickness and lesion scoring of rabbit testicle “Johnsen's scoring system”. Long term of dietary CLA supplementation decreased testicular epithelium thickness at a dose dependent manner (5a) but induced testicular degeneration (5b) in CLA 1% group only.

Lesion scoring results of testicular tissue are presented in **Fig 5.** Testicular epithelial thickness was decreased when CLA dose increased (*P* < 0.01); however, testicular degeneration lesion scoring was significantly affected (i.e., degeneration was increased) by CLA 1% (*P* < 0.01).

**Fig 6:**
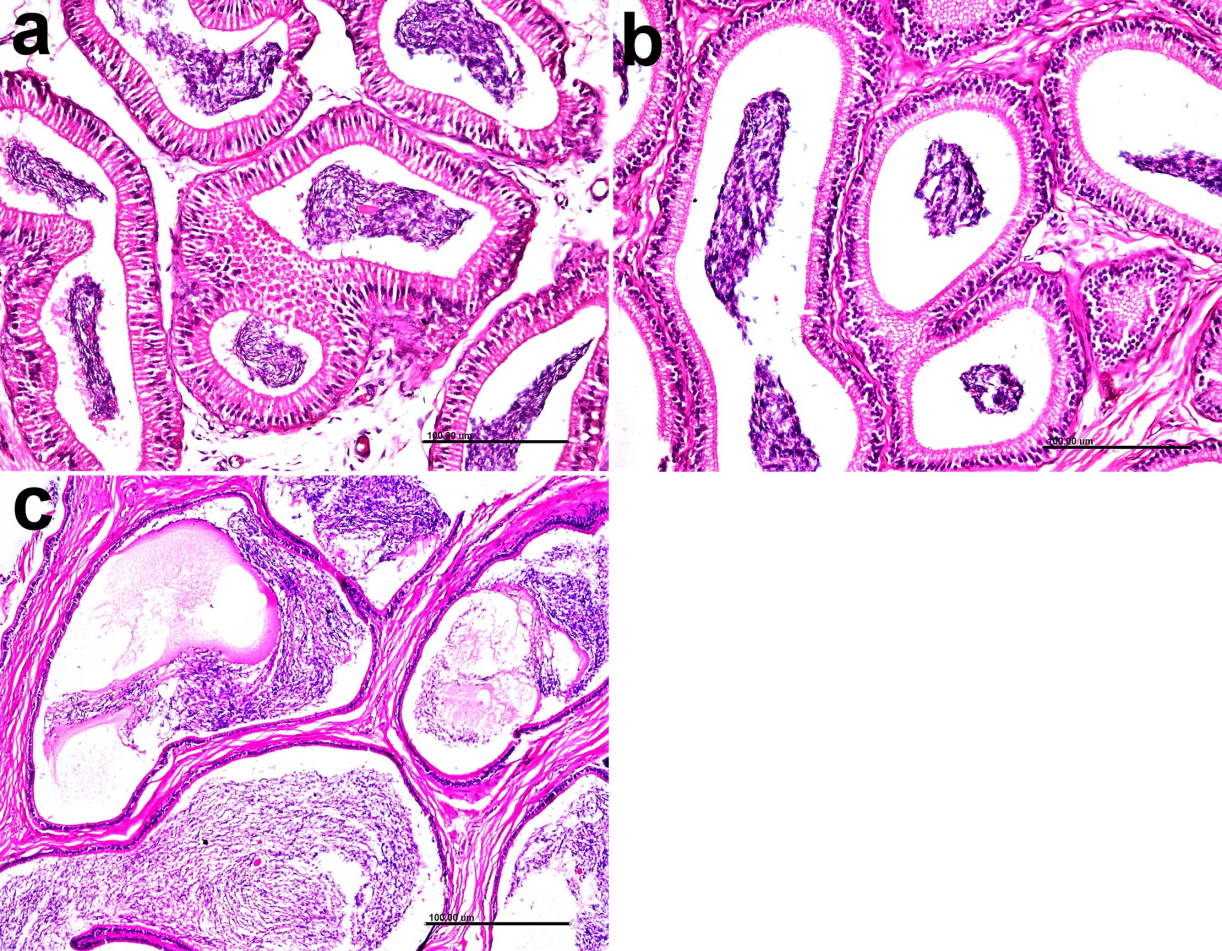
Effect of long-term CLA supplementation on rabbit epididymis. Normal histological structure in CON (6a) and CLA 0.5% (6b) groups, dilation of the epididymal duct with sperm stasis, in the group treated with 1% CLA (6c). Hematoxylin and eosin stain X 200.

Histopathological examination of the epididymis is shown in **Fig 6**. The epididymis of the CON (**Fig 6A**) and CLA 0.5% (**Fig 6B**) groups had normal histological structure, whereas in CLA 1% group there was cystic dilatation of epididymal tubules and sperm stasis in cauda epididymis (**Fig 6C**).

**Fig 7:**
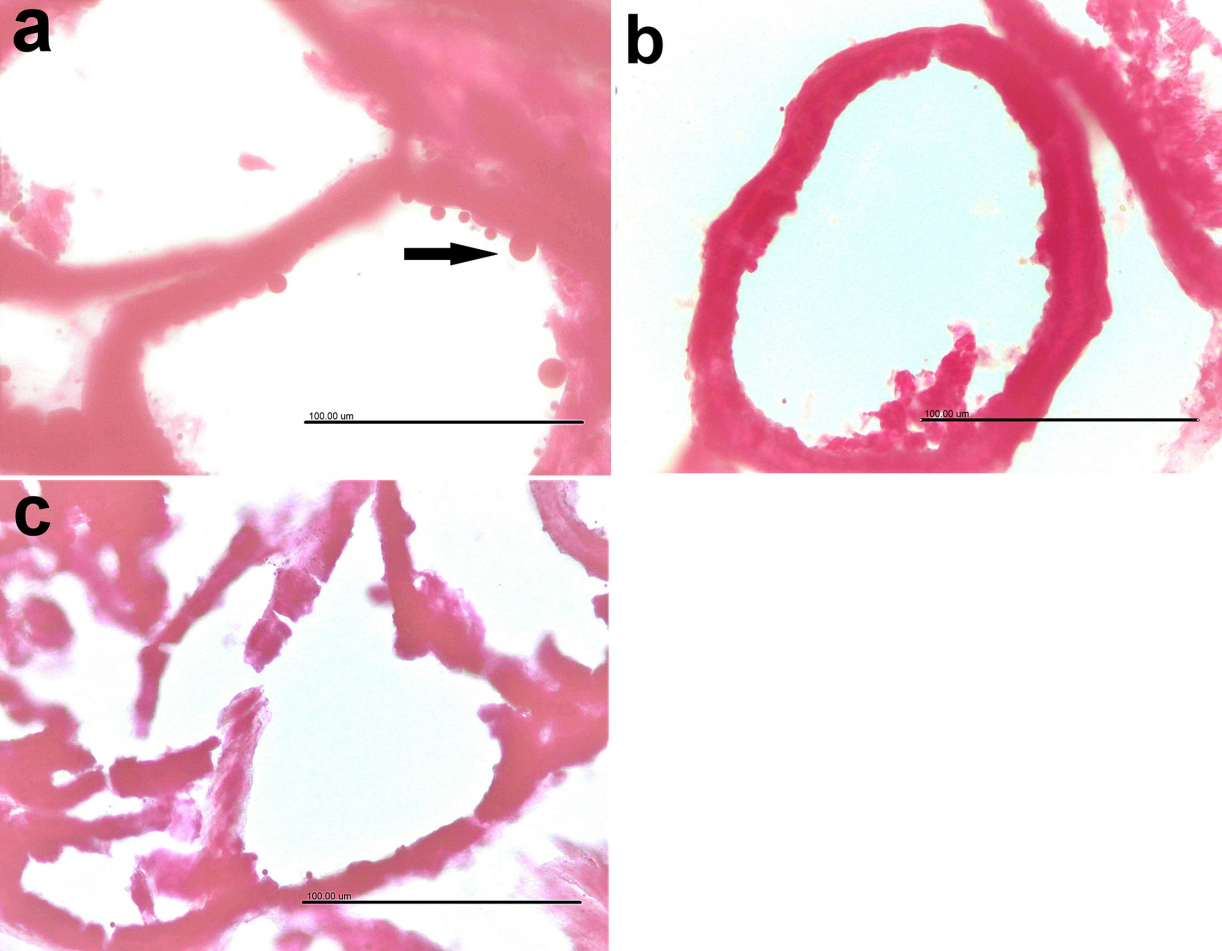
Effect of long-term CLA supplementation on epididymal fat globules “stained with Sudan black”. Presence of fat globules on the surface epithelium of epididymis in the control group (7a), minute fat globules in CLA 0.5% group (7b), and almost diminished in CLA 1% group (7c). Sudan Black X 400.

The staining of the epididymis with Sudan black stain is shown in **Fig 7.** Fat globules were present on the surface of the epithelium of epididymis in the CON group (**Fig 7A**), whereas they were minute in the CLA 0.5% group (**Fig 7B**), and almost absent in the CLA 1% group (**Fig 7C**).

### Immunohistochemistry findings

**Fig 8:**
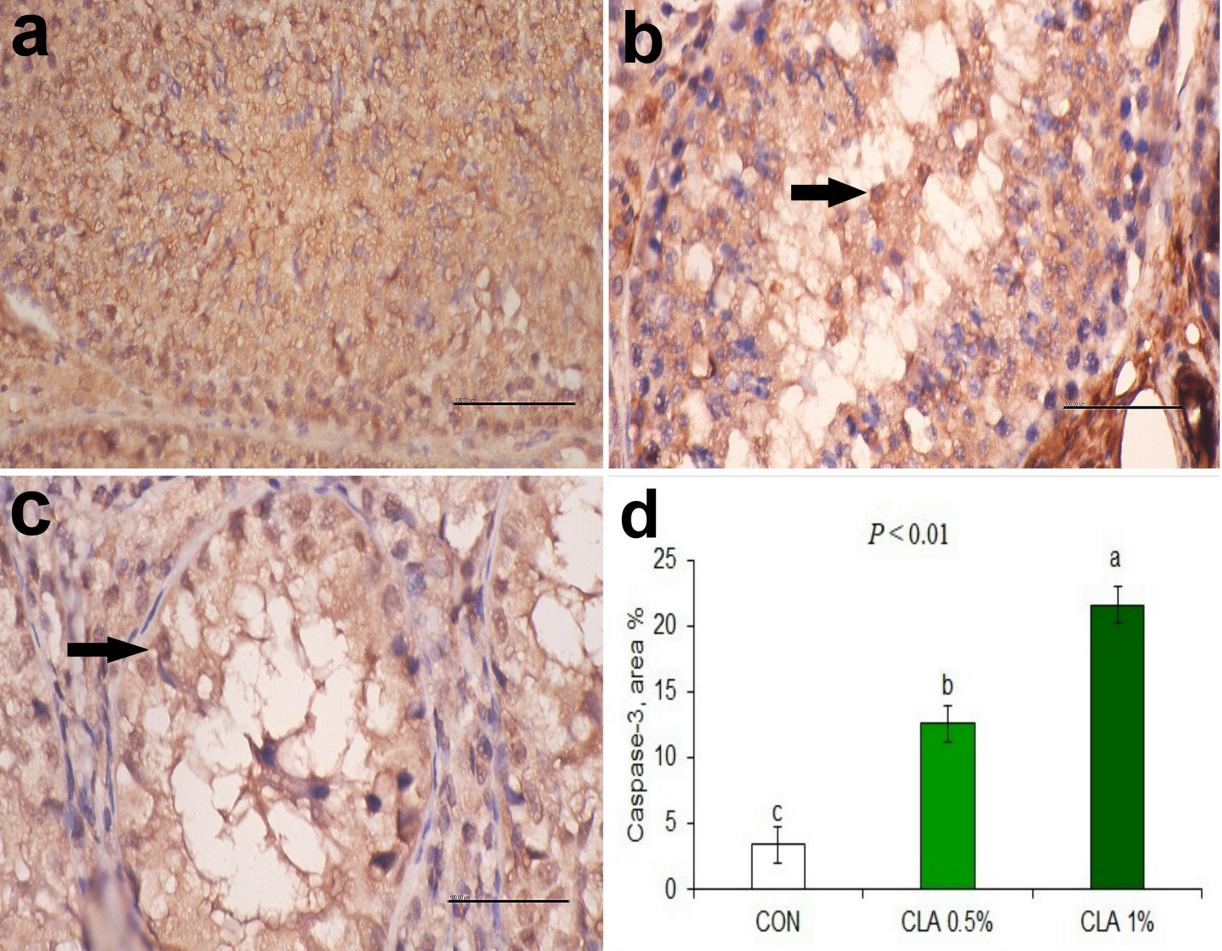
Effect of long-term CLA supplementation on immune histochemistry of Caspase-3 rabbit testicular tissue. Negative staining for caspase-3 in the control group (8a). Brown positively stained spermatogonial cells were observed in the lumen and in the lining epithelium of seminiferous tubules in CLA 0.5% (8b), and CLA 1% (8c) groups. Immuno-peroxidase and hematoxylin counterstain X 400. Area percent of Caspase-3 in different groups, long term dietary supplementation of CLA increased area percentage of Caspase-3 in a dose dependent manner (8d).

The results of caspase-3 staining of testicular tissue are shown in **Fig 8.** The testis of the CON group presented a minimal (< 5%) staining for caspase-3 (**Fig 8A**). On the other hand, brown positively stained spermatogonial cells were observed in the lumen and in the lining epithelium of seminiferous tubules in both CLA 0.5% (**Fig 8B**) and CLA 1% (**Fig 8C**) groups in a dose dependent manner. Area of positive caspase-3 cells was significantly increased when CLA dose increased (**Fig 8D**, *P* < 0.01).

### Transcription of lipid synthesis and apoptosis related genes in testicular tissue and epididymal fat

**Fig 9:**
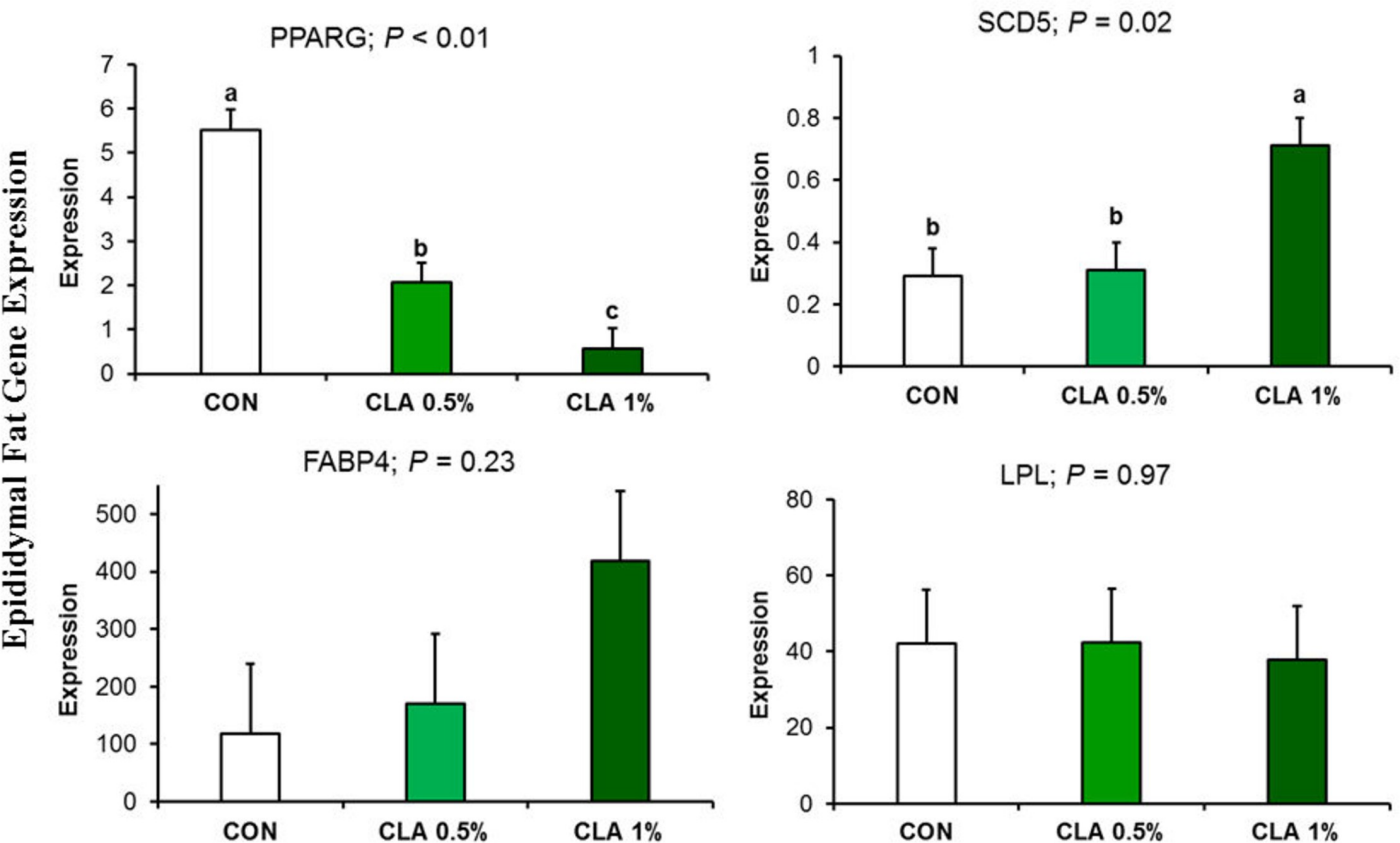
Epididymal fat gene expression in male rabbits receiving long-term CLA supplementation. *SCD5* gene was upregulated in CLA1% group only, while *PPARG* was downregulated in a dose dependent manner. FABP and LPL did not significantly different.

The expression of key lipogenic genes in epididymal fat is shown in **Fig 9.** The expression of *PPARG* was downregulated by CLA in a dose dependent manner (*P* < 0.01). The expression of *SCD5* gene was upregulated in CLA 1% but not in CLA 0.5% (*P* = 0.02).

**Fig 10:**
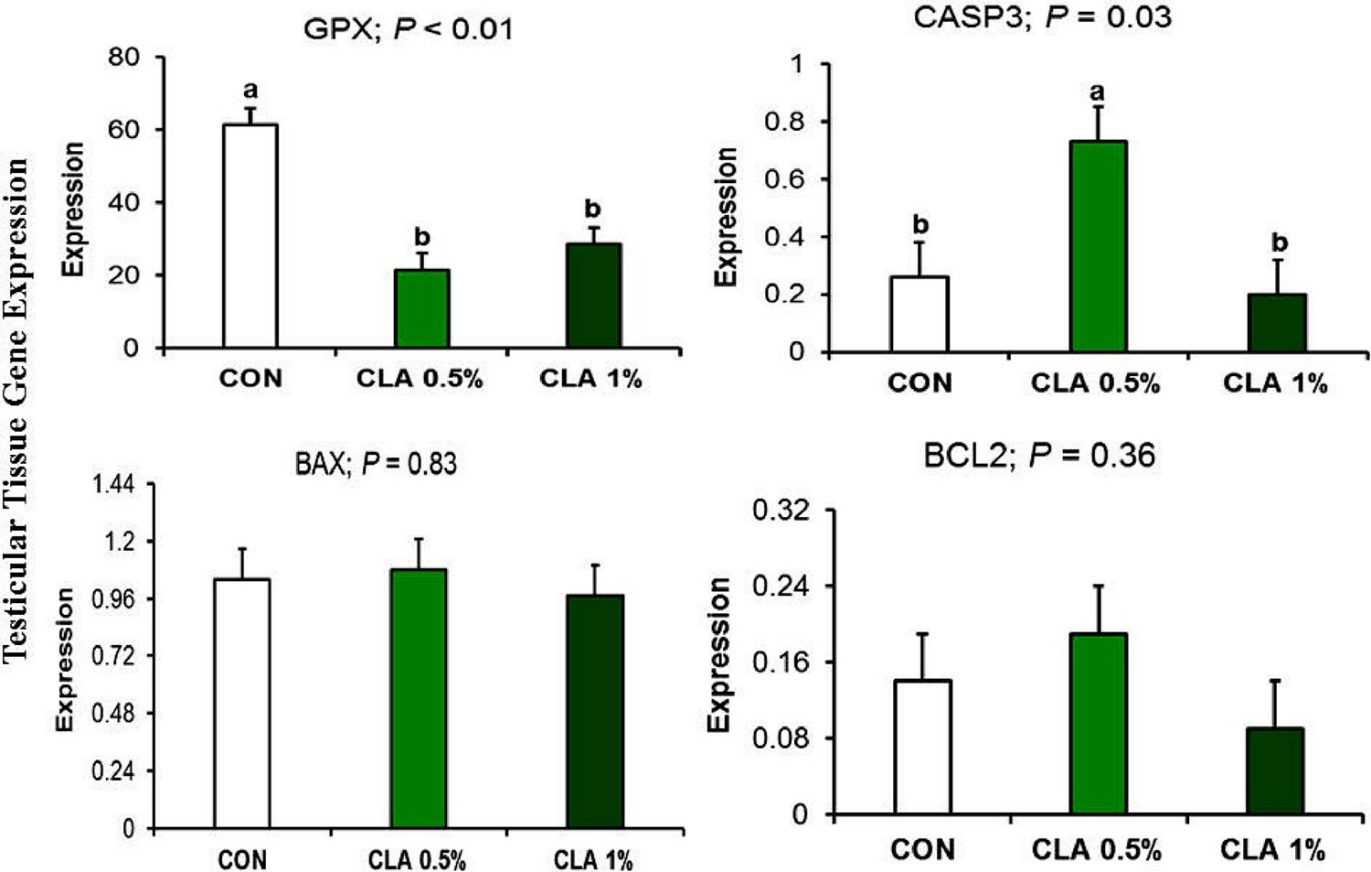
Testicular gene expression in male rabbits fed receiving long-term CLA supplementation. *GPX* gene was down regulated in CLA fed male rabbits, however, *CASP3* gene coding for protein regulating apoptosis was upregulated in CLA 1% group.

The expression of key apoptosis regulatory genes (*CASP3*, *BAX*, and *BCL2*) and the antioxidant-related gene *GPX* in the testicular tissue is shown in **Fig 10.** The expression of the pro-apoptotic *CASP3* gene was increased by CLA 0.5% group but not CLA 1% group (*P* = 0.03). The expression of *GPX* was downregulated in both CLA 0.5% and CLA 1% fed groups (*P* < 0.01).

## Discussion

Several studies were conducted to investigate the growth promoting [36], immune stimulant [37], anti-inflammatory, and anti-carcinogenic effects of CLA [38]. However, studies investigating the long-term effects of CLA on body weight and other body organs and systems like male reproductive system are very limited [39]. In the current study dietary supplementation of CLA for 26 weeks (from weaning till puberty) slightly increased feed intake and body weight similar to previous finding [40]. However, other studies detected opposite effect of CLA on body weight and feed intake [41] or no effect [36,42]. Additionally, longitudinal studies of CLA supplementation revealed also different effects of CLA on body weight and body weight gain, some researchers detected body weight re-gain after supplementation of CLA [43,44], and other researchers observed body weight loss [45,46]. To the authors knowledge there are no longitudinal studies that investigated the effect of CLA on growth rate of male rabbits; however, CLA supplementation for short period (49 d) did not affect body weight and feed intake in growing rabbits [27,47]. The contrasting effects of CLA on body weight and feed intake in the above studies could be explained by the difference in species and the period of CLA supplementation. Our study, which is the first one where CLA was fed for a long period, indicated a significant positive effect of CLA on both feed intake and body weight; however, the increase, especially of body weight, can be considered minimal and probably not important in front of its negative impact on fertility. Moreover, it was previously reported an association between decreased fertility and increased body weight in male rabbits [48].

The effect of long-term dietary CLA supplementation on male rabbit reproduction was not formerly investigated. Prior studies investigating the effect of CLA on semen parameters either through dietary supplementation [12] or direct application on semen [8] were of short term and, for the most part, conducted after puberty [7]. In the current study, long-term dietary supplementation of CLA did not significantly affect sperm kinetics or morphology. However, CLA at a dose of 1% reduced sperm concentration. The negative effect of CLA 1% on sperm progressive motility as well other motility parameters measured, might be due to changes in epididymal histological structure, fatty acid profile, and gene expression. PUFA are a special fatty acid components of sperm plasma membrane [49], they easily undergo lipoperoxidation; thus, presence of powerful antioxidant system to protect them from lipoperoxidative damage is crucial.

Our data suggest that feeding 1% CLA negatively impacted sperm nutrition by reducing epididymal fat PUFA portion and disappearance of fat globules in the epididymis. The reduced PUFA might be consequence also of a higher oxidative status with lower antioxidant capacity, as indicated by the lower vitamin E, L-carnitine, and GPX gene expression and by the higher accumulation of MDA in testicular tissue [49–51]. The increased MDA and downregulation of testicular *GPX* transcript in our study was similar to prior findings [52]. The effect of CLA on regulation of tocopherol is not clear. In short term studies CLA increased liver and adipose tissue tocopherol [52]. However, longer supplementation of CLA during pregnancy and lactation reduced liver tocopherol by 24% in rat dams and increased it in their adipose tissue [53].

The upregulation of *SCD5* in the current study was associated with a decreased PUFA. This finding appears contradictory; however, it has been reported that PUFA inhibit *SCD* activity and transcription [54,55] The downregulation of *PPARG* in this study is similar to prior studies [56,57] and might explain the overall decrease of total epididymal fatty acid, similar to Hue et al., [58].

Supplementation of 1% CLA for 26 weeks tended to reduce the level of arachidonic acid in epididymal fat pad, similar to a prior study [59]. Arachidonic acid is essential for the synthesis of prostaglandin D2, known to inhibit cyclooxygenase-2-induced apoptosis [49].

Leydig cells hyperplasia was also observed in the testicular tissue of group treated with 1% CLA which could be due to hormonal imbalance [60]. A study that was conducted to investigate the effect of CLA on the pituitary-testes axis in rams showed an increased concentration of luteinizing hormone (LH) by CLA [61]; the depletion of LH was associated with Leydig cell atrophy in adult rats [62].

In the present study, the caspase-3 was positively stained in spermatogonial cells of the seminiferous tubules in the groups treated with CLA which demonstrated a dose-related increase in apoptosis [63]. However, on the transcriptional level the transcription of *CASP3* was upregulated only in CLA 0.5% but not in CLA 1%. L-carnitine is known to inhibit the activity of caspases [64]. Thus, the higher level of L-carnitine in CLA 0.5% compared to CLA 1% might explain the higher apoptosis in CLA 1% despite higher *CASP3* expression in CLA 0.5%.

In former reports applying different doses of CLA isomers mixture exerted different effects on several body metabolites [65–67], that could be explained by tissue affinity of CLA, whereas, low dose of CLA affected only retroperitoneal fat depot, while the higher dose affected both retroperitoneal and epididymal fat in mice model [67].

To our knowledge, there are no former studies that showed a positive effect of CLA on apoptosis in the testis although it has been recorded in adipose tissue [68]. Whether the CLA exerts its effect on the testes directly or indirectly is unknown. However, it could be suggested that CLA mediate theirs effect on the testis by affecting the fatty acid composition of epididymal fat with consequent reduction of spermatogenesis [69] and affecting the synthesis of hormones involved in reproduction by disturbing the pituitary-testis axis [70].

## Study limitation

The rabbit number (*n* = 4/group) used in this study was similar to prior studies conducted on rabbits [71–77]. Despite the proper selection of the statistical model, proper sampling and technical replicates analyzed, the low number of animals per group is considered a limitation of the present study.

## Conclusion

Our data suggest that long-term supplementation of CLA is not beneficial to male rabbits, especially if fed with a dose larger than 0.5%. The growth promoting effect might be of interest, but the magnitude does not seem to balance the negative effects on fertility.
