# Correction: Long term conjugated linoleic acid supplementation modestly improved growth performance but induced testicular tissue apoptosis and reduced sperm quality in male rabbit

**DOI:** 10.1371/journal.pone.0231280

**Published:** 2020-03-27

**Authors:** A. M. Abdelatty, O. A. M. Badr, S. A. Mohamed, M. S. Khattab, S. H. M. Dessouki, O. A. A. Farid, A. A. Elolimy, O. G. Sakr, M. A. Elhady, G. M. K. Mehaisen, M. Bionaz

The tenth author's name is spelled incorrectly. The correct name is: G. M. K. Mehaisen.
